# Inherited Biotic Protection in a Neotropical Pioneer Plant

**DOI:** 10.1371/journal.pone.0018071

**Published:** 2011-03-31

**Authors:** Alain Dejean, Bruno Corbara, Céline Leroy, Jacques H. C. Delabie, Vivien Rossi, Régis Céréghino

**Affiliations:** 1 CNRS, Écologie des Forêts de Guyane (UMR-CNRS 8172), Campus Agronomique, Kourou, France; 2 Université de Toulouse, UPS, Toulouse, France; 3 CNRS, UMR 6023, Laboratoire Microorganismes Génome et Environnement (LMGE), Aubière, France; 4 Clermont Université, Université Blaise Pascal, LMGE, Clermont-Ferrand, France; 5 Laboratório de Mirmecología, Convênio UESC-CEPEC, Centro de Pesquisas do Cacau, CEPLAC, Itabuna-BA, Brazil; 6 CIRAD, Écologie des Forêts de Guyane (UMR-CNRS 8172), Campus Agronomique, Kourou, France; 7 CNRS, UMR 5245, EcoLab (Laboratoire d'Ecologie Fonctionnelle), Toulouse, France; 8 Université de Toulouse, UPS, INPT, EcoLab, Toulouse, France; Royal Holloway University of London, United Kingdom

## Abstract

*Chelonanthus alatus* is a bat-pollinated, pioneer Gentianaceae that clusters in patches where still-standing, dried-out stems are interspersed among live individuals. Flowers bear circum-floral nectaries (CFNs) that are attractive to ants, and seed dispersal is both barochorous and anemochorous. Although, in this study, live individuals never sheltered ant colonies, dried-out hollow stems - that can remain standing for 2 years - did. Workers from species nesting in dried-out stems as well as from ground-nesting species exploited the CFNs of live *C. alatus* individuals in the same patches during the daytime, but were absent at night (when bat pollination occurs) on 60.5% of the plants. By visiting the CFNs, the ants indirectly protect the flowers - but not the plant foliage - from herbivorous insects. We show that this protection is provided mostly by species nesting in dried-out stems, predominantly *Pseudomyrmex gracilis*. That dried-out stems remain standing for years and are regularly replaced results in an opportunistic, but stable association where colonies are sheltered by one generation of dead *C. alatus* while the live individuals nearby, belonging to the next generation, provide them with nectar; in turn, the ants protect their flowers from herbivores. We suggest that the investment in wood by *C. alatus* individuals permitting still-standing, dried-out stems to shelter ant colonies constitutes an extended phenotype because foraging workers protect the flowers of live individuals in the same patch. Also, through this process these dried-out stems indirectly favor the reproduction (and so the fitness) of the next generation including both their own offspring and that of their siblings, all adding up to a potential case of inclusive fitness in plants.

## Introduction

It is thought that ants were initially ground-dwelling predators or scavengers and that they adopted an arboreal way of life with the rise of angiosperms by the mid-Eocene ≈50 million years ago [1], [2]. By preying on insects that they discovered on plant foliage while they were foraging, the workers of ground-nesting species probably constituted the first cases of biotic plant protection. Later, tight evolutionary bonds developed between ants and plants. In what is known as a facultative mutualism, plants induce ants to patrol their foliage by producing energy-rich food rewards such as extra-floral nectar (EFN) and food bodies (FBs), reserving proteins for their own metabolism. By providing the ants the fuel with which to patrol, the plants' foliage is protected through the ants' predatory and territorial defense activities [3]. Myrmecophytes, however, live in an obligatory association with only a small number of plant-ants for which they provide a nesting place in pre-existing cavities (domatia) in live plant organs, such as leaf pouches and hollow stems or thorns, and frequently also food (i.e., EFN and/or FBs). In return, plant-ants protect myrmecophytes from several kinds of enemies, particularly defoliating insects [3].

As the basis of most food webs, plants have had to evolve defensive strategies against herbivorous insects. These defenses can be “constitutive” through physical barriers and the continuous production of toxic compounds, or “induced” following attacks by herbivorous insects that trigger the production of defensive chemicals or the emission of volatiles that attract the natural enemies of the attacking insects [4]. Among plant defensive strategies, the biotic, indirect defense provided by ants is of particular interest because herbivorous insects have rarely developed counter-adaptations against ants [5], [6]. Indeed, the positive effects of biotic defense by ants on their host plant's fitness have been unambiguously shown through a meta-analysis [7]–[9].

By concentrating ants on their crowns through the presence there of domatia, myrmecophytes benefit from greater protection if compared with plants bearing EFNs alone. This protection is even better when myrmecophytes also bear only EFN and/or FBs [7], [10]. EFN production can be induced through herbivore damage [6] and, in myrmecophytes, the induced recruitment of nestmates by ants discovering a leaf wound suggests the presence of an induced defense (induced response) [6], [11].

The optimal defense theory predicts that, due to their costs, defenses are deployed in direct proportion to the value and/or risk of the plant parts being attacked. These costs correspond to the production of secondary compounds and/or the formation of mechanical structures which would otherwise be allocated to plant growth and/or reproduction [12]–[14]. In other words, plants invest in constitutive defenses for organs of high value (e.g., reproductive organs, stems) and likelihood of attack (e.g., young parts), while parts of lower value or likelihood of attack (e.g., leaves) are typically protected through induced defenses [15]–[18]. Because of their partnership with ants, many plants bear EFNs not only on their vegetative parts, but also on organs related to reproduction such as inflorescences, sepals, petals, and fruits [18]–[22]. Yet, due to their predatory ability and/or their territorial aggressiveness, ants can attack pollinators, limiting their access to flowers. These ant-pollinator conflicts can disrupt plant reproduction, something particularly true when the EFNs are situated close to flowers. Several processes can attenuate these conflicts such as (1) EFNs distracting ants from floral nectar, (2) flowers attracting pollinators when ants are less active, (3) EFNs active on young plant parts while inflorescences develop on old shoots, and (4) flowers producing pollen repellent to ants [22]–[26].

**Figure 1:**
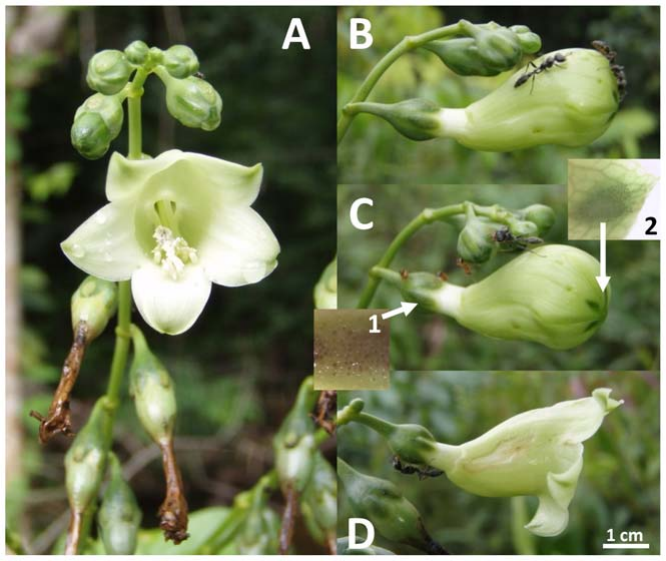
*Chelonanthus alatus* inflorescences showing the different steps from bud formation to withering. A. An inflorescence with an open flower. B. Extremity of an inflorescence with a bud just before blooming. One *Pseudomyrmex gracilis* worker is exploiting the surface of the nectaries situated at the tips of each of the five petals. C. Illustration of the different circum-floral nectaries: (1) on the sepals, and (2) externally on the petals where their tips merge together. An examination of the surface of the nectaries with a stereomicroscope showed numerous stomatal pores where droplets of nectar had been excreted. D. Flower that had begun to wither with a *P. gracilis* worker licking nectar from a sepal. The stomatal pores situated at the tip of the petals are no longer active at this stage.

The focal species of this study, *Chelonanthus alatus* (Gentianaceae), is a Neotropical bi-annual to perennial pioneer geophyte that colonizes both human- and naturally-disturbed sites, as well as inselbergs (i.e., a mountain or rocky mass that has resisted erosion and stands isolated in an essentially level area; also called ‘monadnock’) [22], [27], [28]. The terminal inflorescences bloom year-round and are pollinated by bats [29]. Like for some other bat-pollinated plants, the flowers have petals that do not open completely at anthesis, forming a pseudo-tubular corolla at the base, while the distal part flares into a wide opening (Fig. 1). The sepals of the calyx dorsally bear blunt, thickened, longitudinal keels where ‘circum-floral nectaries’ (CFNs) are located. Like EFNs, CFNs do not play a role in pollination [20]; instead, they attract and retain ants in locations where they can best protect flowers from herbivorous insects. *Chelonanthus alatus* is self-compatible, with seed dispersal by gravity (barochory) or wind (anemochory) [20], [29].

In this study, we hypothesized that *C. alatus* has evolved a relationship with ants such that they protect the plant from herbivorous insects in return for nectar rewards. To test this hypothesis, we first verified the distribution of *C. alatus* individuals that seem dispersed in patches where still-standing, dried-out hollow stems (hereafter “dried-out stems”) are interspersed among live individuals at different stages of development. Second, we verified the lifespan of live *C. alatus* individuals, measured the stem diameter and height of ones that had recently died and verified the longevity of dried-out stems. Third, we compared the ant species visiting the CFNs with the ant community in the areas where *C. alatus* grows. Fourth, we also examined the nest site selection of the ants in the area to verify whether some of them nest in live, hollow *C. alatus* stems and/or in dried-out *C. alatus* stems. Finally, we sought to determine if ants nesting in the dried-out stems protect the surrounding live *C. alatus* plants from herbivores.

## Results

### Formation of *Chelonanthus alatus* patches

**Figure 2:**
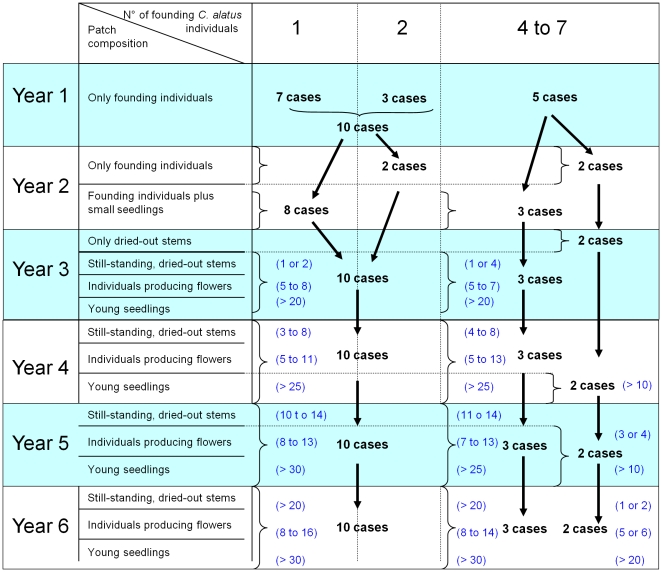
Formation of the *Chelonanthus alatus* patches during a 6-year-long survey. The numbers between parentheses correspond to the numbers of individuals from each case described in the corresponding line of the second column.

**Figure 3:**
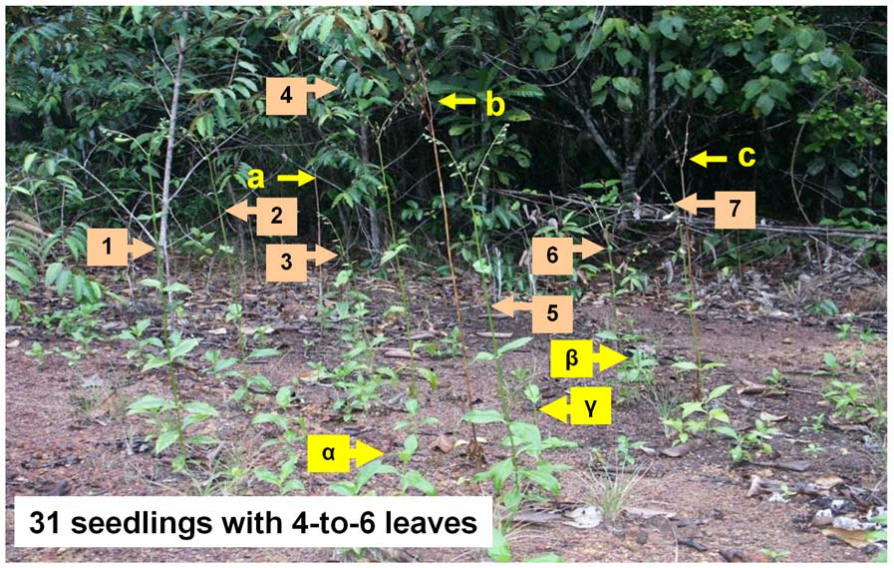
A 4-year-old patch of *Chelonanthus alatus* showing individuals at different stages of development. Three individuals (a, b, c) are dried out, seven others (1, 2 …, 7) are in bloom, three more (α, β, γ) have begun to grow, while the 31 remaining are seedlings with 4-to-6 leaves.

We monitored the changes occurring over 6 years in 15 patches for which we had witnessed the development of the first *C. alatus* individuals in areas that had been recently-cleared (Fig. 2). Seven patches were founded by only one individual plant, the eight others by two to seven individual plants growing in a 3 m radius. Despite this variation in the number of founding plants, in 13 patches the numbers of young seedlings and individuals producing flowers were very similar from the third year (a 4-year-old patch is presented in Fig. 3). Yet, the fate of new seedlings in two other patches was different as only dried-out stems remained after the second year. In that case, a second generation of numerous young seedlings appeared, but only during the fourth year; individuals bearing flowers and fruits appeared during the fifth year (Fig. 2). Given the large numbers of seedlings, they probably originated from the generation of *C. alatus* that had died in the patches rather than from dead individuals from other patches through anemochory.

The formation of patches in recently-cleared areas can be summarized as follows. When one windborne *C. alatus* seed germinates in a favorable area, a ‘founding’ individual develops. Through barochorous seed dispersal, its offspring cluster all around it forming a patch of related *C. alatus* individuals. Then, several generations succeed one another. In all cases, some of the seeds produced can be carried by the wind to a new pioneer area, permitting a new cycle to begin. Concerning pollination, because *C. alatus* is self-compatible and mostly pollinated by bats [20], [29] known to visit open flowers in succession, the opportunities for intra-patch pollination are numerous and help to maintain relatedness between individuals. Yet, inter-patch pollination also occurs as nectarivorous bats can travel over relatively long distances [29].

### Lifespan of *Chelonanthus alatus* and longevity of dried-out stems

Among the 15 live *C. alatus* individuals tagged in January 2001, 11 lived for 18 months and then died during the dry season, and the remaining four were still alive 2 years later (i.e., 3.5 years from the beginning of the survey). Among the dried-out stems tagged at the beginning of the experiment, six decayed after 12–18 months during their second rainy season. The same fate was noted for the 11 dried-out stems corresponding to the individuals from the previous survey that died after 18 months. The other nine remained upright during the 2-year survey; the diameter of their stems was significantly larger than those that decayed faster (means±SE; 0.8±0.04 cm *vs.* 0.45±0.03 cm, respectively; df = 23; t = 6.07; P<0.0001).

**Figure 4:**
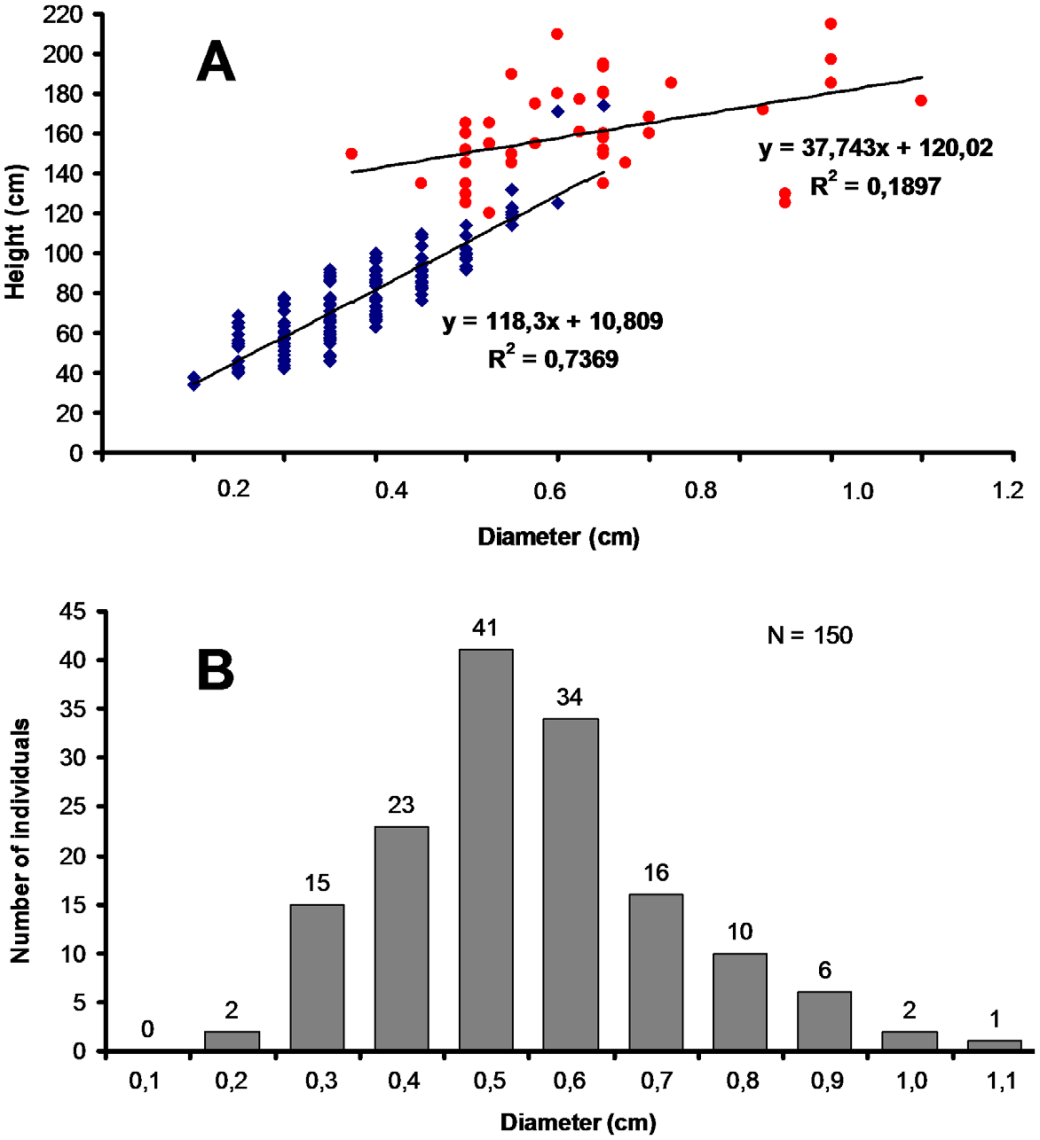
Stem diameter and height of *Chelonanthus alatus*. A. Blue diamonds correspond to the relationship between the stem diameter and height of 150 *C. alatus* individuals that had recently died (note that these individuals had lost their leaves and were turning brown; the diameter of their stems, taken 5 cm from the ground, will vary only very slightly as they will dry and will remain still standing). Red circles correspond to 53 isolated, founding *C. alatus* individuals. Note that both their stem diameter and height are higher than those from the patches (see text for statistics). B. Distribution of the number of individuals based on the diameter of their stems.

By measuring the stem diameter and height of 150 *C. alatus* individuals that had recently died, we were able to establish a relationship curve between these two variables (Fig. 4A). If compared to the measurements of the *C. alatus* that had died earlier, those that produced persistent, dried-out stems (i.e., 0.8±0.04 cm in diameter) were, based on this curve, among the tallest individuals (see also Fig. 4B). Also, the stem diameters of 150 *C. alatus* individuals that had recently died were significantly smaller than those of 90 dried-out stems selected at random and for which we do not know how much time separated the measurement from the death of the plant (means±SE; 0.55±0.01 cm *vs*. 0.61±0.02 cm; t = 2.44; df = 238; P<0.05). We can therefore distinguish small individuals with a short lifespan from taller individuals with a longer lifespan and larger diameter at their base.

We also monitored 53 founding *C. alatus* individuals at the stage when they were turning brown. They had a wider stem diameter and were taller than the 150 individuals from the previous lot (Fig. 4A) (means±SE; stem diameter: 1.053±0.037 cm *vs*. 0.55±0.01 cm; t = 15.68; df = 201; P<0.0001; height: 159.8±3.2 cm *vs*. 76.0±1.9 cm; t = 22.39; df = 201; P<0.0001).

### Ant diversity and activity

We found no ants inside any of the 98 live stems recorded in the 6-year-old patches surveyed. In contrast, 114 of the 214 (i.e., 53%) dried-out stems contained ants. Occupied stems had significantly larger diameters than uninhabited, dried-out stems (0.52±0.02 versus 0.30±0.01; df = 208; t = 9.24; P<0.0001). Compared to the diameters of plants that had recently died (Fig. 4A), dried-out, ant-inhabited stems correspond to medium to large-sized *C. alatus* individuals.

Of the 44 ant species inventoried during this study, as expected, most are species characteristic of perturbed habitats. Among these ants, none nested in live *C. alatus* individuals; six nested in the hollow, above-ground parts of dried-out stems (predominantly *Pseudomyrmex gracilis* found in 83 of the 114 inhabited, dried-out stems; 73%; Table 1) and 12 in the base (e.g., less than 5 cm high) of these dried-out stems (mostly ground-nesting species with tiny workers expanding their colony to the root area); 20 were recorded in the surrounding plant foliage; and 26 were captured using pit-fall traps. We noted low Sørensen similarity indices between ant species related to *C. alatus* and those recorded on adjacent trees or captured using pit-fall traps (Table 1).

**Table 1:** Ants inhabiting dried-out, hollow *Chelonanthus alatus* stems among those noted in the area studied.

				Nested in the dry *C. alatus* stems (% of 114 cases)		Exploited *C. alatus* EFNs (% of 80 cases)		Noted on adjacent trees	Captured in pit-fall traps (% of 40 cases)
No. species	SF	Nest.	Ant species	Base	aerial parts	diurnally	nocturnally		
1	M	G	*Atta sexdens*						2.5
2	D	A	*Azteca bequaerti*					**+**	
3	D	A	*Azteca chartifex*					**+**	
4	F	M	*Brachymyrmex* sp. 1	2.6		18.75	11.25	**+**	
5	F		*Camponotus* sp. 1			1.25			10.0
6	F	G	*Camponotus blandus*			100.00		**+**	10.0
7	F		*Camponotus crassus*					**+**	
8	F	A	*Camponotus femoratus*					**+**	
9	F	A	*Camponotus latangulus*		0.9	1.25		**+**	2.5
10	F	G	*Camponotus melanoticus*				32.50	**+**	5.0
11	F		*Camponotus novogranadensis*					**+**	
12	M	G	*Cardiocondyla obscurior*	1.75					
13	M	A	*Crematogaster* sp. 1		4.4	15.00	7.50	**+**	7.5
14	M		*Crematogaster* sp. 2						2.5
15	M		*Crematogaster* sp. 5					**+**	
16	M		*Crematogaster* sp. 9						2.5
17	D	G	*Dorymyrmex pyramicus guy.*						7.5
18	E	G	*Ectatomma brunneum*			3.75		**+**	15.0
19	E	G	*Ectatomma tuberculatum*			15.00	11.25	**+**	
20	F	G	*Gigantiops destructor*			1.25		**+**	2.5
21	P	G	*Hypoponera opaciceps*	1.75					
22	M	G	*Nesomyrmex tristani*	5.3					
23	D		*Linepithema* sp.					**+**	
24	P	G	*Odontomachus caelatus*						2.5
25	P	G	*Pachycondyla mesonotalis*		0.9				
26	F		*Nylanderia* sp. 1	3.5		1.25			2.5
27	F		*Nylanderia* sp. 2	0.9					15.0
28	F		*Nylanderia* sp. 3	0.9					
29	M	G	*Pheidole fallax*			15.00	21.25		7.5
30	M		*Pheidole* sp. 4						2.5
31	M		*Pheidole* sp. 9						17.5
32	M		*Pheidole* sp. 29						10.0
33	M	G	*Pheidole* sp. 30	0.9		2.50			
34	M		*Pheidole* sp. 31					**+**	7.5
35	M		*Pheidole* sp. 37						7.5
36	Ps	A	*Pseudomyrmex ethicus*		0.9	1.25			
37	Ps	A	*Pseudomyrmex gracilis*		73.0	100.00		**+**	7.5
38	Ps	A	*Pseudomyrmex* sp. gr. *pallidus*		5.3	7.50		**+**	2.5
39	Ps	G	*Pseudomyrmex termitarius*	2.6*		7.50		**+**	12.5
40	M	G	*Solenopsis saevissima*			1.25			12.5
41	M	G	*Solenopsis* sp. 1	0.9					
42	M	G	*Strumigenys louisianae*						2.5
43	M	G	*Wasmannia auropunctata*	0.9		3.75		**+**	10.0
44	M	G	*Wasmannia* sp.	0.9					
			Number of ant species	12	6	17	5	20	26
			No. ant species (EFNs pooled)	12	6	18		20	26
			Sørensen sim. Ind. Pit-fall traps	0.21	0.25	0.64		0.43	-
			on adjacent trees	0.18	0.31	0.63		-	-
			on *C. alatus* EFNs	0.33	0.42	-		-	-
			Base *versus* hollow stems	0.00		-		-	-

Note: List of ant species living inside of 144 dried-out, hollow *Chelonanthus alatus* stems; foraging on *C. alatus*, or on adjacent trees; and recorded inside of 18 pit-fall traps. A total of 44 ant species recorded. Subfamilies (SF) = D: Dolichoderinae; E: Ectatomminae; F: Formicinae; M: Myrmicinae; P: Ponerinae; Ps: Pseudomyrmecinae. Nesting habit (Nest.) = G: ground-nesting species; A: arboreal species; M: generalist able to nest in different situations. * only incipient colonies. For EFNs, the percentages were obtained from the presence of workers of corresponding species on at least one plant in the patch (eight patches; 10 series of observations).

Ants visited the CFNs situated on the sepals from the beginning of the formation of the buds until the formation of the fruits. They also visited the surface of the nectaries situated externally at the tip of each petal that are active only prior to the opening of the flowers (Fig. 1B–C).

**Figure 5:**
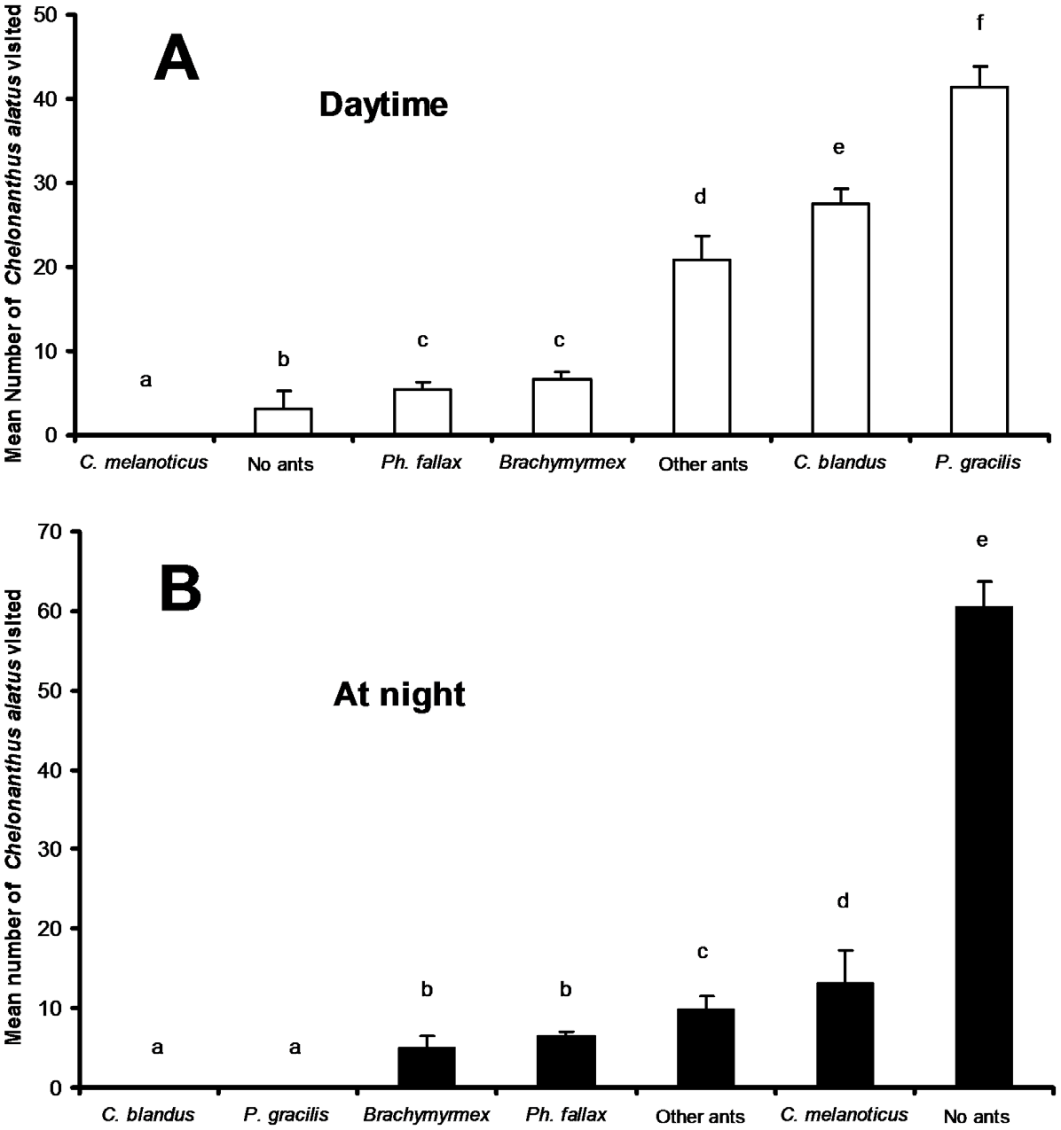
Ant visitation of *Chelonanthus alatus*. Mean (±SD) number of *C. alatus* individuals visited by ants (or individuals not visited) for their CFNs (98 plants surveyed; 10 non-consecutive days of observation). A: during the daytime (3 hours of observation per day during two periods: 10:00–11:00 and 17:00–18:00). B: At night (one 2-hour period of observation per night: 23:00–01:00). Statistical comparisons. One-way analyses of variance during the daytime: F = 580.4; P<0.0001 and at night: F = 414.5; P<0.0001; Newman-Keuls' *post-hoc* tests: different letters indicate significant differences at P<0.05 between the daytime and at night.

Five of the six ant species nesting in the above-ground parts of the dried-out stems exploited the CFNs of live *C. alatus* in the same patch, and therefore situated in the vicinity (namely, *Camponotus latangulus*, *Crematogaster* sp.1, *Pseudomyrmex ethicus*, *P*. *gracilis*, and *Pseudomyrmex* sp.,gr *pallidus*). This was also the case for five of the 12 species nesting in the base of the dried-out stems (namely, *Brachymyrmex* sp., *Nylanderia* sp. 1, *Pheidole* sp. 30, *Pseudomyrmex termitarius*, and *Wasmannia auropunctata*) and for seven ground-nesting species (namely, *Camponotus blandus*, *Camponotus melanoticus*, *Ectatomma brunneum*, *Ectatomma tuberculatum*, *Gigantiops destructor*, *Pheidole fallax*, and *Solenopsis saevissima*; see also the Sørensen similarity index; Table 1). *Camponotus blandus* (Formicinae) and *P. gracilis* (Pseudomyrmicinae) workers were the most numerous diurnally, exploiting the CFNs of several *C. alatus* in all of the patches, while the other 15 species recorded were much less numerous. Nocturnally, the CFNs were exploited by only five species of which *Camponotus melanoticus* pre-dominated (Table 1). By scoring the number of times the ants visited the CFNs per *C. alatus* individual, we noted that during the daytime *P. gracilis* workers were the most frequent, followed by *C. blandus*. At night, *C. melanoticus* pre-dominated as previously noted, but 60.5% of the *C. alatus* individuals were not visited by ants, which was unusual during the daytime (Fig. 5).

We recorded between five and 30 dried-out *C. alatus* sheltering *P. gracilis* individuals per patch (10.38±8.4 on average), corresponding to 82 and 411 workers, respectively (143±111 workers per patch on average; 8 patches). In total, of the 83 dried-out stems sheltering *P. gracilis*, four contained an incipient colony (i.e., a queen, up to 4 ‘small’ workers and brood), while the 79 others sheltered 14.3±3.7 workers plus abundant brood; the queens were present in only one to four dried-out stems per patch.

### Plant protection

Observations conducted during 10 non-consecutive days on 98 live *C. alatus* individuals from eight patches revealed that the inflorescences were attacked by cockroaches (diurnally in 60 cases; nocturnally in 179 cases), and by curculionid and chrysomelid beetles (diurnally in 25 cases; nocturnally in 23 cases). Adjusted to the 12 h/12 h distribution of the nycthemeron, the number of observations per day and the 10 days of observation, this corresponds to a total of ca. 0.35 daily visits by defoliating insects per inflorescence during the daytime and ca. 2.47 at night.

Of the more than 500 live *C. alatus* observed in total during preliminary experiments and during this study, the foliage of only one individual had been attacked by caterpillars. Concerning hemipterans, colonization by Coccidae attended by *Crematogaster* sp.2 workers was noted once, while other cases corresponded to isolated individuals (Cicadellidae: four times; Fulgoridae: once; Membracidae: twice).

We also conducted an experiment comparing *C. alatus* individuals bearing flowers from unaltered patches (control) with those from two experimental treatments. The first experimental treatment corresponded to patches where we had torn out all of the dried-out stems to eliminate their ant inhabitants, and so their anti-defoliator activity. In the second experimental treatment, we spread a ring of Tanglefoot® at the base of the plants to prevent any ants from climbing up (including species nesting in the ground and in nearby dried-out stems plus those coming from neighboring areas).

**Figure 6:**
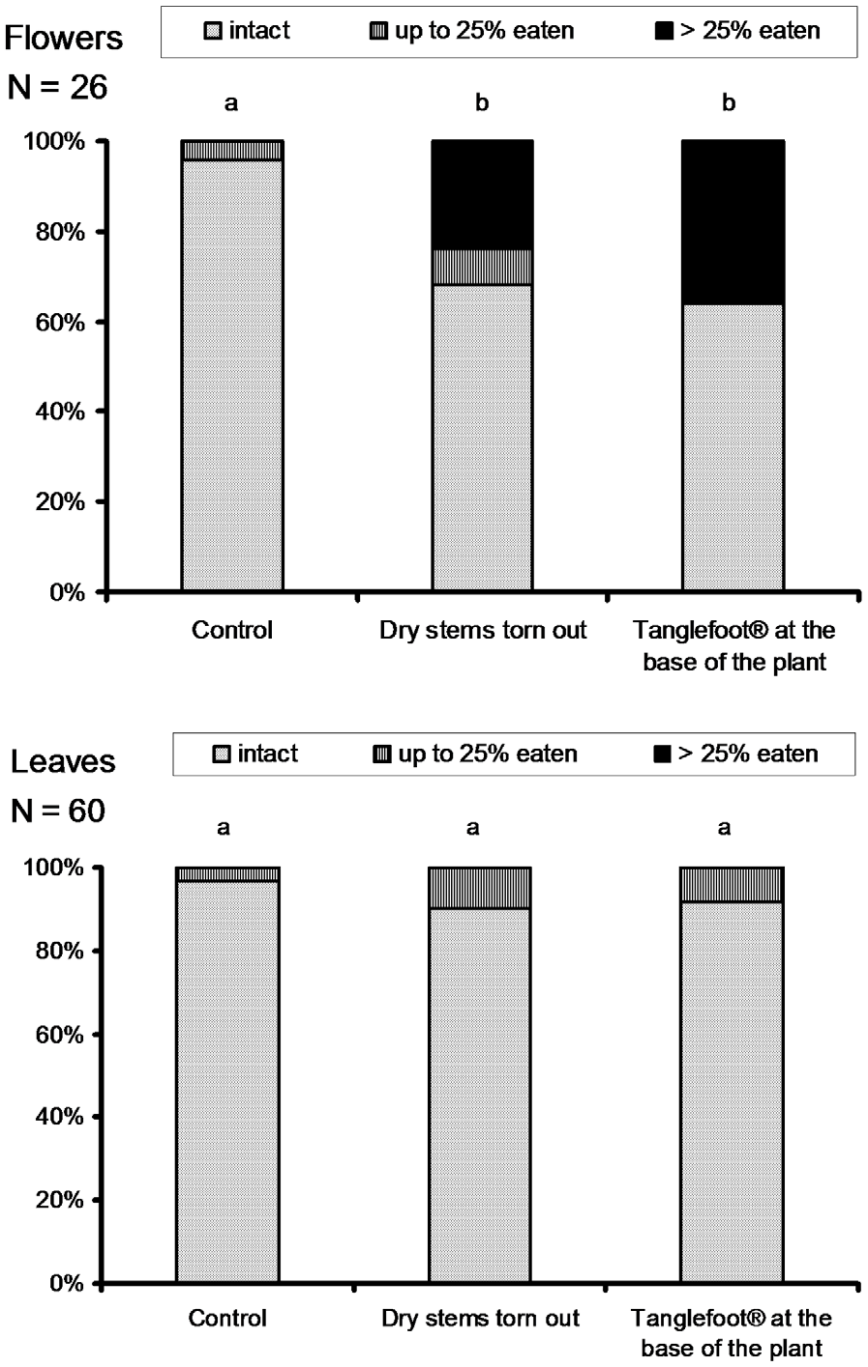
Ant protection of *Chelonanthus alatus*. Percentages of petal (Flowers) and leaf (Leaves) surface destroyed by defoliating insects in three situations. Control: unaltered patches; first experimental treatment: individuals from patches whose standing, dried-out stems were all torn out (eliminating their ant inhabitants); second experimental treatment: individuals for which a ring of Tanglefoot® was spread around their base to prevent any ant from climbing up. Statistical comparisons (Likelihood ratio test for nested models): different letters indicate significant differences at P<0.05. Because we surveyed the two last, opposite leaves, the statistics were calculated from the 30 pairs.

We modeled the rate at which the petals of flowers were attacked by defoliating insects with a generalized linear model (GLM) using an ordinal probit link function of the treatment. The experimental treatments had a significant effect on the rate at which the petals were attacked by defoliating insects (Likelihood ratio test: P = 0.006); the difference between the two experimental treatments was not significant (Likelihood ratio test: P = 0.4) (Fig. 6). In other words, the rate at which the petals were attacked by defoliating insects was significantly lower for the *C. alatus* in the unaltered patches than for those from either experimental treatment. Thus, it is likely that much of the flower protection was provided by ants, mostly *P. gracilis*, nesting in dried-out stems.

Yet, the ants appear to provide only weak biotic protection to the foliage as we saw no significant difference in the percentage of foliar surface eaten by insects between plants from unaltered patches and those from either experimental treatment (Likelihood ratio test: P = 0.25; Fig. 6).

## Discussion

This study indicates the existence of a facultative mutualism between live *C. alatus* individuals and the workers of various opportunistic ant species that visit their CFNs and/or forage for prey on their foliage. Among them, *P. gracilis*, *C. blandus* (both diurnal) and *C. melanoticus* (nocturnal) pre-dominated (Fig. 1; Table 1). These species were also recorded in the same habitat exploiting the EFNs of *Passiflora* spp. [30].

The circum-floral position of these nectaries encourages ants to actively defend the reproductive - but apparently not the vegetative - *C. alatus* tissues (see [19] for a similar case for a Mexican orchid). This is consistent with the optimal defense theory predicting that plants invest in the defense of parts with a high fitness value, such as reproductive organs [12]–[18]. Yet, concerning the foliage, the plants' anti-insect compounds seem sufficient (see [31] for secondary compounds in Gentianaceae), so that biotic protection was not demonstrated and the rate of defoliation was low in all cases. This can explain why we noted only a few cases of hemipterans attacking *C. alatus*.

Also, ant-pollinator conflicts, which can disrupt plant reproduction [22]–[26], seem to be resolved in this case as the flowers attract pollinators when the ants are less active (see also [24]). Indeed, it is unlikely that *C. melanoticus* workers perturbed bat pollination because 60.5% of the plants' inflorescences were not visited by ants at night (Fig. 1), whereas nuptial nectar production is mostly nocturnal [29]. Furthermore, the pollinating bat, *Glossophaga soricina*, very common in French Guiana, is also insectivorous, and its hovering visits are extremely short [29], [32].

Although *C. blandus* and *C. melanoticus* also visited the CFNs, the case of *P. gracilis* merits particular attention as its workers were by far the most frequently noted. Known as the “twig-ant”, this species nests in dried-out, hollow twigs into which the workers are able to gnaw entrance holes [33], [34]. In this study, *P. gracilis* colonies, known to be polydomous (multiple nests) and polygynous (multiple queens) [35]–[37], nested in several dried-out *C. alatus* stems, and each patch probably contained only one colony (confrontation tests; unpublished results). The workers are territorial and are known to be efficient predators [36]–[38], attacking other ants experimentally placed on their host plant [34]; they can even prey on other ant species (see Fig 1C).

Concerning the plant, by spreading a ring of Tanglefoot® at the base of *C. alatus* individuals, we showed that, in general, the ants protect the flowers, but not the vegetative tissues. Because very similar results were obtained by eliminating only those ant species nesting in dried-out stems (that were torn out), we can deduce that the latter, mostly *P. gracilis*, account for most of the protection of the *C. alatus* flowers. We also noted that dried-out stems sheltering ant colonies can persist for several years thanks to their structure (they are typically tall, long-lived *C. alatus* individuals), the quality of the wood that contains fungicides [39], and the anti-termite action of the ants nesting in their base.

Consequently, although it corresponds to a facultative mutualism, this situation is similar to that involving myrmecophytes and plant-ants. Indeed, *C. alatus* likely derives a benefit in terms of fitness because its flowers are protected, while furnishing food (CFNs) and a favorable nesting site to a limited number of ant species, mostly *P. gracilis*. Yet, the hollow, dried-out internodes lodging ants, related to the phenology of *C. alatus*, are not real ‘ant-domatia’ based on the following definition which applies to the live parts of plants: “plant structures that appear to be specific adaptations for ant occupation, often formed by the hypertrophy of internal tissue at particular locations on the plant, creating internal cavities attractive to ants” [40]. Although they may be located in hollow thorns or leaf petioles, in leaf pouches and on fruits, most ant domatia are caulinary; that is to say, live hollow stems and shoots [6], [10]. The facultative but persistent associations noted in the present study suggest an evolving mutualism, and can shed light on how such interactions might develop over evolutionary timescales. Indeed, another case of a non-myrmecophytic plant sheltering ants in persistent, hollow structures related to the plant's phenology - here inflorescence production - has been reported in the Araceae [41].

Therefore, we suggest that the character “still standing, dried-out hollow stems” favors – through facultatively mutualistic ants - the reproduction (and so the fitness) of both their own offspring and those of their siblings, all of which corresponds to a case of inclusive fitness in plants. Inclusive fitness can be defined as the adaptive value (fitness) of an individual, taking into account not only that individual's own reproductive success, but also the success of its entire kin (i.e., those bearing some portion of the same genotype [42]). The development of the founding *C. alatus* individuals improves the ecological niche for future generations through the persistence of their dried-out stems that provide a nesting site for colonies of a facultative, mutualistic ant. Then, some individuals from each generation bequeath this improvement to the next generation. Because the *C. alatus* genes are expressed beyond their immediate boundaries through these persistent dried-out stems, one can consider that this example corresponds to an “extended phenotype” [43] rather than “niche construction” which implies that genes are not involved in the legacy [44] (see also the controversy on this subject in [45]).

## Materials and Methods

### Study site

This study was conducted between 2001 and 2010 in French Guiana near the Petit Saut dam (5°03′39″N, 53°02′36″W). Surveys on the formation of the *C. alatus* patches and on the relationship between stem diameter and plant height were conducted along the last 15 km of the road leading to the dam, plus the areas of *Keren Roch* and *Base vie* situated 0.4 km and 1 km from the dam, respectively. The other surveys were conducted on individuals forming patches along the dirt road leading to *Crique Plomb* constituting a narrow, cleared area situated in the middle of the rainforest.

The research undertaken meets all applicable standards for the ethics of experimentation and research integrity.

### Formation of *Chelonanthus alatus* patches

Between 2001 and 2002, we registered the formation of 15 new *C. alatus* patches in different, recently-cleared areas. In each case, we noted the number of individuals and mapped them. Then, we noted the fate of the formation of these patches over 6 years, recording three times a year the numbers of young seedlings, individuals bearing flowers and still-standing, dried-out stems.

### Lifespan of *C. alatus* individuals and longevity of dried-out stems

In January 2001, we tagged 15 young individuals bearing four leaves and 15 stems on dead plants starting to turn brown in order to know the lifespan of *C. alatus* individuals and why the dried-out stems do not decay, but remain standing in the patches. We verified if they were still standing every 6 months during 2 years. Using calipers, we measured the diameter 5 cm from the ground of all of the stems at the beginning of the study; using the unpaired t-test, we compared the diameter of the stems that had dried-out and decayed 12–18 months later with those that remained standing.

We also measured the stem diameter and height of (*i*) 150 *C. alatus* individuals that had recently died to establish a relationship curve between these two variables as well as (*ii*) 90 “old” dried-out stems selected at random, and (*iii*) 53 founding *C. alatus* individuals that had also recently died. Using the unpaired t-test, we compared the first two lots to know if still-standing, dried-out stems are among the tallest and/or the widest; then the first and the third lots to know if founding individuals are taller and have a wider diameter than individuals chosen at random. Because the first lot was compared twice, probabilities were adjusted using the sequential Bonferroni procedure.

### Ant diversity and activity

In order to know which ants species are present in eight *C. alatus* patches (2.5-to-5 m×2 m) as well as in the surrounding vegetation over a 2-m-wide area bordering each side of each patch, we did the following. First, we placed five pit-fall traps in each patch for 24 h (a total of 40 pit-fall traps) as it has been demonstrated that the data gathered through the use of 20 pit-fall traps is robust enough to characterize a habitat in French Guiana. This method also permits the comparison of sites whose habitat is disturbed to different degrees (see [46]). We then conducted two periods of observation during the daytime (10:00–11:00 and 17:00–18:00), and another at night (23:00–01:00) for 10 non-consecutive days (five observers). These periods of observation were chosen because they correspond to the major periods of activity of diurnal and nocturnal ant species, respectively, in this area (see [47], [48]).

We also noted which ant species visited the CFNs of each *C. alatus* plant. Finally, we collected the ants sheltering in the hollow stems of all of the *C. alatus* from the eight patches, including dried-out individuals, by cutting them at their base and putting each plant into a separate plastic bag; we then transported everything to the laboratory.

We used the Sørensen similarity index to compare the ant species visiting different plants or patches because it gives low weights to outlier values (see Table 1). In the Sørensen similarity index (QS = 2C/A+B), A and B are the number of species recorded in samples A and B, respectively, and C is the number of species shared by the two samples.

We compared the number of *C. alatus* individuals visited diurnally and nocturnally for their CFNs by the different ant species using a one-way ANOVA followed by a Newman-Keuls *post-hoc* test for multiple comparisons.

In order to know if ants nest randomly in the dried-out stems or if they rather select wide individuals, using the unpaired t-test, we also compared the diameter of 127 uninhabited stems with 83 others sheltering ants.

Voucher specimens of the ants were deposited in the *Laboratório de Mirmecologia* (CPDC collection, CEPEC-CEPLAC, Itabuna, Bahia, Brazil).

### Plant protection

We verified the impact of the ants on *C. alatus* flowers and leaves by comparing the percentage of surface eaten by defoliating insects for three groups of 30 *C. alatus* plants (55–70 cm tall) bearing flowers. The objective of the experiment was to eliminate the possibility for ants to protect live *C. alatus* from defoliators through their predatory and/or their territorial behavior. Our experimental design included three treatments: unaltered patches (control) and two experimental treatments. Each of these three treatments were included in each of three different patches (i.e., 3×3 = 9 patches in total) along 700 m of the *Crique Plomb* dirt road. In the first experimental treatment, we tore out all of the dried-out stems, thus eliminating their ant inhabitants (mostly *Pseudomyrmex gracilis*). So, ground-nesting species plus those from the neighboring areas were free to exploit the *C. alatus* CFNs. In the second experimental treatment, we spread a ring of Tanglefoot® at the base of the stems of live individuals to prevent any ants from climbing up. Because Tanglefoot® is toxic for plants, we first rolled a 5 cm wide band of aluminum foil around the base of the stem, and then deposited the Tanglefoot® on the aluminum.

We used the two youngest leaves on each *C. alatus* (total of 30 pairs of leaves in each of the three replicates: control and the two experimental treatments; i.e., 30×3 = 90 pairs of leaves assessed) and verified the percentages of leaf surface destroyed after 20 days following the start of the experiment (at which time, both the flowers and the leaves were intact). Due to their short lifespan, we obtained only 26 flowers (each from a separate plant individual) from each of the three replicates. The experiment lasted 5 days, starting before the buds were ready to open (see Fig 1B) until the flowers began to wither.

We defined three rates of attack: (1) not at all attacked; (2) up to 25% of the petals or leaf surface destroyed; and (3) more than 25% of the petals or leaf surface destroyed.

The results were analyzed using an ordinal regression since the rate of attack was an ordinal response. The relationship between the rate of attack of a flower (or a leaf) and the treatment was modeled with a GLM using a probit link [49]. The link function was selected, among the usual adapted link functions for ordinal data, according to the Akaike Information Criterion. To avoid confusion due to an eventual micro-site effect, we alternatively attributed treatments to the nine patches: control treatment, first treatment, second treatment, control treatment, etc. We verified the homogeneity of this experimental design and did not detect a ‘patch effect’ (Likelihood ratio tests; P = 0.9 for the flowers and P = 0.74 for the leaves).

Statistical analyses were conducted using GraphPad Prism 4.03, Inc. software and R 2.10.1 software [50].
